# Puerarin-loaded PEG-PE micelles with enhanced anti-apoptotic effect and better pharmacokinetic profile

**DOI:** 10.1080/10717544.2018.1455763

**Published:** 2018-03-28

**Authors:** Wenqun Li, Junyong Wu, Jiang Zhang, Jingjing Wang, Daxiong Xiang, Shilin Luo, Jianhe Li, Xinyi Liu

**Affiliations:** a Department of Pharmacy, The Second Xiangya Hospital, Central South University, Changsha, China;; b Institute of Clinical Pharmacy, Central South University, Changsha, China;; c College of Pharmacy, Changsha Medical College, Changsha, China;; d School of Pharmaceutical Sciences, Central South University, Changsha, China

**Keywords:** PEG-PE micelles, hemolysis, pharmacokinetics, cellular uptake, apoptosis

## Abstract

Puerarin (PUE) is the most abundant isoflavonoid in kudzu root. It is widely used as a therapeutic agent for the treatment of cardiovascular diseases. However, the short elimination half-life, poor-bioavailability, and acute intravascular hemolysis of PUE are the main obstacles to its widespread clinical applications. Whereas PEG-PE micelles possess the ability to release medicine slowly, enhance the cellular uptake of drugs and improve their biocompatibility. Therefore, it was aim to fabricate puerarin-loaded PEG-PE (PUE@PEG-PE) micelles to improve the pharmaceutical properties of drugs. It can be observed from the TEM images that PUE@PEG-PE micelles appeared obvious core-shell structure and remained well-dispersed without aggregation and adhesion. PUE was successfully embedded in the core of PEG-PE micelles, which was confirmed by FT-IR and ^1^H NMR spectra. *In vitro* studies showed that PUE@PEG-PE micelles exhibited a sustained release behavior in pH 7.4 PBS buffer and decreased hemolysis rate of PUE. Compared with PUE, PUE@PEG-PE micelles showed a 3.2-fold increase in the half-life of PUE and a 1.58-fold increase in bioavailability. In addition, the PUE@PEG-PE micelles exerted enhanced protective effect against isoprenaline-induced H9c2 cells apoptosis compared with PUE, as evident by decreased percentage of Hoechst-positive cells, Caspase 3 activity, Bax expression, and increased Bcl-2 expression. Notably, the PEG-PE micelles exhibited favorable cellular uptake efficiency on H9c2 cells, and this may account for their enhanced anti-apoptotic effect of the incorporated drug. Altogether, the PUE@PEG-PE micelles were not only able to control the drug release but also offered promise to enhance the pharmacokinetic and pharmacodynamic potential of PUE.

## Introduction

Puerarin (PUE) is the major bioactive ingredient in kudzu root (Yuan et al., 2014). Previous researchers show that PUE has anti-apoptotic (Liu et al., 2012, 2013), anti-oxidant (Liu et al., 2011), anti-inflammatory (Huang et al., 2012), anti-arrhythmic (Zhang et al., 2011), and anti-fibrosis (Lu et al., 2014) activity. For example, Liu et al. demonstrated that anti-apoptosis and anti-hypertrophy effects of PUE on isoprenaline (ISO)-induced H9c2 cells were observed in an *in vitro* study (Liu et al., 2015). A recent study also found that PUE pretreatment protected the H9c2 cells against daunorubicin-induced cytotoxicity by inhibiting cell apoptosis as evidenced by decreased expression of cleaved caspase 3 (Li et al., 2017). Research on the clinical application of PUE injection in recent years indicates that PUE has been approved by the State Food and Drug Administration in China for clinical therapy in the treatment of cardiovascular diseases (Wong et al., 2011). Owing to short elimination half-life of PUE, frequent injection is often needed. The clinical results show that some patients may experience severe and acute side effects which appear after several weeks of therapy (Wu et al., 2014). In particular, the acute intravascular hemolysis (AIH) of PUE injection is the most serious, for it can be fatal. The mechanism underlying PUE-induced hemolysis remains uncertain but may be partly related to high concentrations of PUE resulted from frequent dosing (Luo et al., 2011). In 2011, Hou et al. reported the interaction between high concentration PUE and erythrocyte membrane in certain solution, resulting in membrane disorganization and, eventually, cytolysis (Hou et al., 2011). Increase in drug circulation time may improve the therapeutic effect and potentially reduces the overall dose required, resulting in avoiding the likelihood of AIH due to high doses of PUE. Therefore, it is necessary to develop a sustained-release drug system to prolong the drug action time, reduce the adverse reaction of high plasma concentration and improve patient compliance so as to achieve favorable therapeutic goals.

In the current study, 1,2-Distearoyl-*sn*-glycero-3-phosphoethanolamine-*N*-[methoxy(polyethylene glycol)-2000] (PEG-PE) micelles are widely used in novel drug delivery vehicles, they are formed through the self-assembly of amphiphilic polymers in aqueous media (Sarisozen et al., 2012b). The hydrophilic PEG_2000_ groups that form the shell of the micelles are responsible for reducing the uptake of the reticuloendothelial system (RES), which results in the prolonged circulation of the micelles *in vivo* (Sarisozen et al., 2012a), while the highly hydrophobic PE residues of the micelle core can solubilize hydrophobic drugs (Sawant et al., 2008). Moreover, non-immunogenic surface coatings of PEG have excellent biocompatibility and ‘invisibility’ properties (Yassin et al., 2015), the dense PEG corona in micelles provide a protective shield for the entrapped molecules, reducing the chances of the drug being recognized by T cells and macrophages *in vivo* (Lazarjani et al*.,*^2010^). Therefore, the PEG-PE micelles hinder interactions with blood components, reduce non-specific adhesion of plasma proteins and increase blood circulation time (Endres et al., 2011). In addition, PEG-PE micelles may be used to protect the embedded molecules from degradation in circulation and also have the potential to slowly release the drug molecules over a period of time due to their long fatty acyl chains, which confer less mobility to the entrapped drug, thus prolonging their circulation time (Dabholkar et al., 2006).

Recently, it is reported that PEG-PE micelles have great potential for drug delivery due to prolonged circulation time (Sawant & Torchilin, 2010), enhanced trans-membrane transport of drugs and altered drug internalization route and subcellular localization properties (Kohay et al., 2017). In 2012, Wang et al. demonstrated that the encapsulation of anti-cancer drugs in PEG-PE micelles was accelerated to enter cells resorting to the increased membrane fluidity and permeability exerted by PEG-PE insertion, increasing their cytotoxicity *in vitro* and enhancing their anti-tumor activity *in vivo* (Wang et al., 2012). The increased anti-tumor effect of micelles-encapsulated drugs *in vitro* is largely due to their enhanced cellular accumulation and uptake without altering the drug's mechanism (Lu et al., 2010). In addition to small molecule drugs, PEG-PE micelles could also be able to deliver biomacromolecules such as DNA and RNA. It was reported recently that the siRNA-loaded PEG-PE micelle had a transfection efficiency 50-fold higher than that of naked siRNA (Musacchio et al., 2010). Therefore, based on the strong trans-membrane transportability of PEG-PE micelles, they have great potential to enhance the pharmacological effects of drugs.

In this study, we prepared PUE-loaded PEG-PE (PUE@PEG-PE) micelles, using PE as a core and PEG chains for the shell. Based on their excellent properties such as high stability, good biocompatibility, extended circulation time, and enhanced cellular uptake, the PUE@PEG-PE micelles were expected to perform prolonged *t*_1/2,_ improved AUC_0–∞_values in the pharmacokinetic analysis and enhanced anti-apoptotic activity in H9c2 cell.

## Materials and methods

### Materials

1,2-Distearoyl-sn-glycero-3-phosphoethanolamine-N-[methoxy(polyethyleneglycol)-2000] (PEG_2000_-PE) was acquired from Lipoid GmbH (Ludwigshafen, Germany). PUE and its injection were purchased from Shandong Fangming Pharmaceutical Group Co., Ltd (Shandong, China). Coumarin-6 (C6) and isoprenaline (ISO) were purchased from J&K Chemical Ltd (Shanghai, china) and 4'6-diamidino-2-phenylindole (DAPI) was provided by Beyotime Biotech (Jiangsu, China). H9c2 cells derived from rat myocardium were purchased from the American Type Culture Collection (Manassas, VA). Cell cultures were maintained in a humidified atmosphere of 5% CO_2_ at 37 °C. All other chemicals and reagents were analytical grade or chromatography grade.

### Preparation of drug-loaded micelles

PUE@PEG-PE micelles were prepared by the solvent evaporation method. The PEG_2000_-PE (100 mg) and PUE (8 mg) were co-dissolved in 20 mL of methanol. The solvent was dried under reduced pressure in a round-bottom flask at 37 °C. Almost immediately the obtained thin film was kept in vacuum for over 6 h to remove any traces of remaining solvent. Then, the thin film was hydrated in 5 mL of distilled water at 37 °C, cooled to room temperature and filtrated through 0.22 μm Millipore filter to afford a clear solution and remove undissolved drug and copolymer. Spontaneous formation of micelles was initially confirmed by the observation of a transparent reddish solution and Tyndall phenomenon. To study the cellular uptake of PEG-PE micelles by H9c2 cells, the coumarin-6 (fluorescent material) loaded PEG-PE micelles (C6@PEG-PE) were prepared by the same way.

### Micelles size, zeta potential and transmission electron microscopy (TEM)

The mean hydrodynamic size and zeta potential were determined by dynamic light scattering (DLS) using a Zetasizer Nano ZS90 instrument (Malvern, UK). The measurement was done in triplicates. For transmission electron microscopy (TEM) (JEM 2100F, Japan) analysis, a single drop of aqueous micelles suspension syringe was placed on a carbon-coated film of 200-mesh copper grid and was air-dried for 10 min. TEM images were acquired at an accelerating voltage of 200 kV.

### Entrapment efficiency (EE) and drug loading (DL)

The loading amount of PUE in PEG-PE micelles was determined by LC-20AT HPLC system equipped with a UV detector set at a wavelength of 250 nm. Prior to assay, the developed micelle formulation was diluted to an appropriate concentration with methanol. Drug loading efficiency (DL%) and entrapment efficiency (EE%) were calculated by the following equations.

$$\text{EE(\%)=}\frac{\text{Weight}\;\text{of}\;\text{drug}\;\text{in}\;\text{micelles}}{\text{Initial}\;\text{weight}\;\text{of}\;\text{drug}}\times \text{100\%}$$

$$\text{DL}(\%)=\frac{\text{Weight}\;\text{of}\;\text{drug}\;\text{in}\;\text{micelles}}{\text{Weight}\;\text{of}\;\text{micelles}\;\text{containing}}\times 100\%$$

### Nuclear magnetic resonance spectroscopic studies (NMR)

In order to confirm the core-corona structure of PUE@PEG-PE micelles, NMR studies were performed. The ^1^H NMR spectra were recorded on a JEOL Eclipse NMR spectrometer operating 400 MHz NMR spectra of PUE in DMSO, blank PEG-PE in D_2_O, and lyophilized PUE@PEG-PE micelles in D_2_O or DMSO.

### Fourier transform infrared spectrometer (FT-IR)

A Thermo Fisher Nicolet iS10 Fourier transform infrared spectrometer was also used to characterize the status of PUE in polymeric micelles. First, a background spectrum of a blank KBr was run. Then PUE was dispersed in KBr pellets. For empty blank micelles or PUE@PEG-PE micelles, the sample was gently coated on the KBr tablets. The FT-IR spectrum of them from 400 to 4000 cm^−1^ was recorded by way of the KBr pellet method on an FT-IR spectrometer at room temperature.

### Micelles stability studies

The PUE@PEG-PE micelles were stored as a micelles suspension at pH 7.4 PBS for 6 weeks at 4 °C, or as a lyophilized powder at −20 °C for 2 months. The stability of the micelles was monitored by the changes in particle size and drug content in the samples during the storage period. Moreover, the serum stability of PUE@PEG-PE micelles was investigated in the presence of 10% FBS. The changes in particle size at 0, 4, 8, 12, and 24 h were monitored by DLS.

### *In vitro* release kinetics

The *in vitro* release study of PUE from drug-loaded micelles was performed via dialysis technology. In briefly, free PUE (100 μg) or PUE@PEG-PE micelles (corresponding to 100 μg PUE) were added into the dialysis bags (MWCO =2000 Da, Spectrum Labs Inc., Rancho Dominguez, CA) and submerged in 40 mL of PBS (pH 7.4) at 37 °C with stirring at 100 rpm for 72 h. At set time intervals, 0.5 mL of the release medium was taken out and analyzed by the HPLC method. The removed solution was immediately replaced with an equal amount of fresh medium.

### Hemolytic assay

Fresh human blood samples were kindly donated by the second Xiangya hospital of the central south university (Hunan, China). Human red blood cells (RBCs) were centrifuged at 2500 rpm for 10 min at 4 °C and further washed more than six times with sterile PBS. The purified RBCs were diluted 10-fold with PBS buffer, and 150 μL of diluted RBCs suspension was added to a 150 μL of PUE and PUE@PEG-PE micelles in PBS (pH 7.4) at a concentration of 12.5, 25, 50, 100, 200, 400, 800 or 1600 μg/mL. PBS and distilled water were used as a negative and positive control, respectively. The vortex-mixed solution was incubated at 37 °C for 4 h, followed by centrifugation at 3000 rpm for 3 min. A total of 120 μL of supernatant from the sample was transferred to a 96-well plate. The absorbance values of the supernatants at 540 nm were measured using a Multiskan MK3 microplate reader. The absorbance of the supernatant at 540 nm was compared with two control samples in order to determine the percentage of hemolysis. The absorbance of the supernatant in PBS was taken as zero hemolysis (0%) and the total hemolysis (100%) was assigned when PBS was replaced by distilled water. The degree of hemolysis was determined by the following equation.

$$\text{Hemolytic}\;\text{ratio }(\%)\;=\;\frac{\text{Sample}\;\text{absorbance}-\text{Negative}\;\text{control}}{\text{Positive}\;\text{control}-\text{Negative}\;\text{control}}$$

### Pharmacokinetic study

The animal experiment was approved by the Animal Ethics Committee at the Institute of clinical pharmacy, Central South University. Male Sprague–Dawley rats (230–250 g) were used and supplied by Hunan Slack Scene of Laboratory Animal Co., Ltd. The rats were treated with PUE and PUE@PEG-PE micelles at a PUE dose of 20 mg/kg by tail vein injection under mild ether anesthesia (*n* = 5 per group). At designated time points after dosing, blood samples (0.4 mL) were collected from the retro-orbital plexus of rats under light ether anesthesia into microcentrifuge tubes containing heparin as anti-coagulant at 0, 5, 15, 30, 45, 60, 120, 180, 240, 360, and 420 min post-dosing, and then centrifuged at 3500 rpm for 10 min. The harvested supernatant plasma was stored at −80 °C until analysis. The plasma sample preparation of PUE was as follows. To 100 μL of plasma sample was added 20 μL of tectoridin (interior label) solution. Following the addition of 400 μL of methanol, the sample was vortex-mixed for 5 min and then centrifuged at 13,000 rpm for 10 min. The resultant supernatant was evaporated to dryness in a water bath at 40 °C under the protection of nitrogen. The dried residue was reconstituted in 40 μL of methanol and vortex-mixed for 1 min. After centrifugation at 10,000 rpm for 5 min, the supernatant was transferred to an autosampler vial, and a 30 μL aliquot of the sample was injected into HPLC system. The chromatographic separation was performed on a Phenomenex Luna C_18_ column (4.6 mm ×250 mm, 5 μm) at a column temperature of 35 °C. The mobile phase consisted of (A) 0.1% aqueous phosphoric acid and (B) acetonitrile using a gradient elution of 12–45% B at 0–6 min, 45–65% B at 6–7 min, 65–12% B at 7–11 min, and the re-equilibration time of gradient elution was 2 min. The flow rate was 1.0 mL/min. Detection was performed at 250 nm.

### Cell culture and treatment

H9c2 cells were cultured in Dulbecco’s Modified Eagle medium (Gibco BRL Co. Ltd., SanFrancisco, CA) supplemented with 10% fetal bovine serum (Gibco BRL Co. Ltd.) in a humidified atmosphere of 5% CO_2_ at 37 °C. The medium was replenished every 2 days. H9c2 cells were divided into four groups as follows: (i) control, (ii) ISO, cells were treated with ISO (10 μM) for 24 h, (iii) ISO + PUE, cells were pre-incubated with PUE (20 μM) for 0.5 h before treated with ISO (10 μM) for 24 h, and (iv) ISO + PUE@PEG-PE, cells were pre-incubated with PUE@PEG-PE (20 μM, PUE equivalent) micelles for 0.5 h before treated with ISO (10 μM) for 24 h. All assays were performed in triplicate and repeated on three separately initiated cultures.

### Hoechst staining

H9c2 cells were seeded on sterile cover glasses placed in 24-well plates and cultured in Dulbecco’s Modified Eagle medium. Upon reaching 50–60% confluence, cells were treated with the above drugs (ISO, ISO + PUE, and ISO + PUE@PEG-PE). After treatment for 24 h, cells were fixed, washed twice with PBS, and stained with Hoechst 33258 staining solution according to the manufacturer’s instructions (Beyotime, Haimen, Jiangsu, China). Image capture and slide evaluations were performed using a Nikon 80i fluorescence microscope equipped with ACT-2 U imaging Software (Nikon, Tokyo, Japan). Apoptotic cells were defined by the condensation of nuclear chromatin, fragmentation, or margination to the nuclear membrane.

### Caspase 3 activity assay

The activity of caspase 3 was determined by using a caspase 3 activity kit (Beyotime Institute of Biotechnology, China), which is based on the ability of caspase 3 to change Ac-DEVDpNA into the yellow formazan product, *p*-nitroaniline (pNA). In brief, cells were resuspended in lysis buffer and left on ice for 10 min. The lysate was centrifuged at 20,000 g for 10 min at 4 °C, and supernatants were incubated with 10 μL caspase 3 substrate (Ac-DEVDpNA, 2 mM) in the dark at 37 °C for 1 h. The release of pNA was qualified with Multiskan Spectrum (Thermo, Waltham, MA) at 405 nm. Caspase 3 activity was expressed as the fold of enzyme activity compared to that of synchronized cells.

### Western blotting

Proteins, extracted from cells with RIPA buffer, were separated by 10% SDS/PAGE and then transferred to polyvinylidene fluoride membranes. Membranes were blocked with milk solution, followed by incubation with primary antibodies overnight at 4 °C. The blots were then incubated with horseradish peroxidase (HRP)-coupled goat anti-mouse or goat anti-rabbit secondary antibody (sc-2005, 1:2000, sc-2030, 1:5000; Santa Cruz, CA). The chemiluminescence signals were detected with the EasySee Western Blot Kit (Beijing TransGen Biotech, Beijing, China). Image J 1.43 (National Institutes of Health, Bethesda, MD) was used for densitometric analysis. Primary antibodies are shown as follows: anti-Bcl-2 (ab692, 1:1000, Abcam), anti-Bax (ab32503, 1:1000, Abcam) and anti-GAPDH (ab9484, 1:1000, Abcam).

### Cellular uptake experiment

C6 was generally used as a fluorescence probe incorporated into nano-sized particles for observation of cellular uptake. H9c2 cells were seeded into 12-well culture plates at 1.0 × 10^5^ cells per well and cultured for 24 h. Then incubated with C6@PEG-PE micelles or C6 solution at an equivalent C6 concentration (0.1 μg/mL) in complete DMEM. After 2 and 4 h incubation, cells were washed with PBS (pH 7.4) three times to eliminate residual C6 outside the cells. After rinsing with PBS, the cell nuclei were stained with DAPI. Fluorescence intensities of C6 inside the cells were observed under fluorescence microscope qualitatively. C6 and DAPI showed green and blue colorations, respectively.

### Intracellular drug intake

To study the cellular uptake of PUE@PEG-PE micelles by H9c2 cells, the initial cell treatment was similar to the above cellular uptake experiment of C6@PEG-PE micelles. H9c2 cells were incubated with PUE@PEG-PE micelles or free PUE solution at an equivalent PUE concentration (20 μM) in complete DMEM. After 2 and 4 h incubation, 100 μL of medium was drawn. After centrifugation at 15,000 rpm for 5 min, the supernatant was transferred to an autosampler vial, and a 10 μL aliquot of the sample was injected into HPLC system. The chromatographic separation was performed on a Phenomenex Luna C18 column (4.6 mm × 250 mm, 5 μm) at a column temperature of 35 °C. The mobile phase consisted of (A) 0.2% aqueous phosphoric acid and (B) acetonitrile using a gradient elution of 14–25% B at 0–6 min, 25–30% B at 6–7 min, 30–14% B at 7–11 min, and the re-equilibration time of gradient elution was 4 min. The flow rate was 1.0 mL/min. Detection was performed at 250 nm.

### Statistical analysis

The data was shown as the mean ± standard derivation. Pharmacokinetics parameters were obtained using the Drug and Statistics (DAS) Version 3.3 software. Student’s *t* test was used to assess differences and correlation between the two groups.

Comparisons among multiple groups were assessed for significance using one-way analysis of variance (ANOVA). The statistical significance for all tests was at *p < .05 and **p < .01.

## Results and discussion

### Micelle formation, nanoparticle size, zeta potential, and morphology

**Figure 1.**
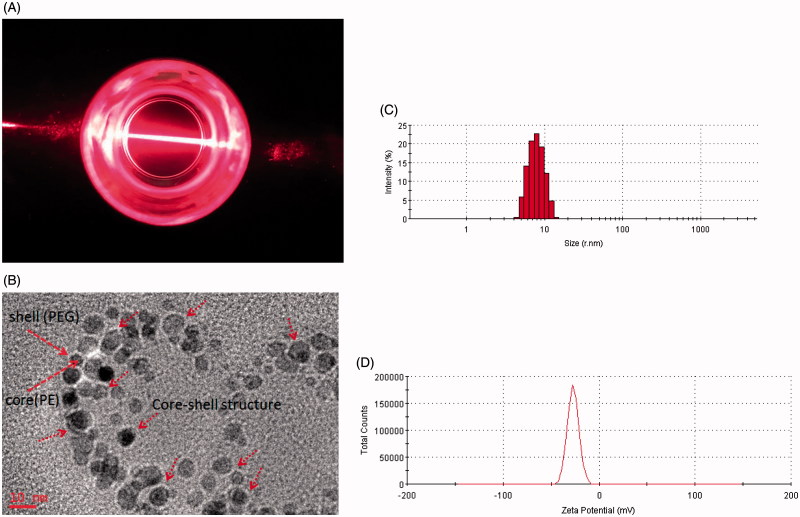
Characterization of PUE@PEG-PE micelles. (A) Transparent reddish solution and Tyndall phenomenon. (B) TEM photographs. (C) Size distribution. (D) Zeta potential distribution.

Amphiphatic polymer could self-assemble to form micelles in an aqueous solution by filming-rehydration method. A transparent reddish solution and Tyndall phenomenon is shown in Figure 1(A) indicated that micelles were formed. Under TEM, The morphology of PUE@PEG-PE micelles was illustrated in Figure 1(B), presenting perfectly spheroidal shape and approximately core–shell structure with small size (Tong et al., 2011). This might be due to the interparticle electrostatic repulsion which may prevent the aggregation of the vesicles and increase the stability of the dispersions (Hu et al., 2014; Szilagyi et al., 2014). Average size of blank PEG-PE micelles was 14.2 nm. PUE@PEG-PE micelles presented in Figure 1(C) were found to be in size range of 16.0 nm. The particle size of drug-loaded micelles was basically consistent with that of empty micelles, which showed that the structure of PEG-PE micelles was not disrupted by encapsulating PUE. From another perspective, the hydrophobic interactions among the PE moiety hold drugs together to form spherical micelles with small size (Chen et al., 2016). In addition, the size of this micelles was large enough to avoid being filtered out by kidneys or absorbed and metabolized by liver, yet small enough to evade the macrophage engulfment in the RES (Wang et al., 2014). Therefore, the PUE@PEG-PE micelles with the particle size of 16 nm could avoid rapid metabolism and elimination and guarantee the stability and long circulation time (Raza et al., 2016). Besides, zeta potential of blank PEG-PE micelles and PUE loaded micelles was −27 and −24.1 eV (Figure 1(D)), respectively. It has been reported that the anionic surface of the nanosystem has better biocompatibility than the cationic surface (Cui et al., 2012).

### Entrapment efficiency (EE%), drug loading (DL%), and in vitro stability

The DL% and EE% for PUE@PEG-PE micelles were around 5.5 and 68.7%, respectively. PUE@PEG-PE micelles remained stable when stored as micelle suspension at 4 °C for 6 weeks and as a freeze-dried powder for 2 months at −20 °C. Neither decrease of drug content nor micelle size distribution changes were observed during the storage period for either the micelle suspension or the micelle powder after rehydration. This high drug loading efficiency and favorable stability of PUE@PEG-PE micelles were attributed to the hydrophobicity of the drugs and the two fatty acid acyls of these micelles in solubilizing and enclosing such lipophilic drugs together (Abouzeid et al., 2014). Thus, PUE could not easily escape into the aqueous phase during the self-assembling process. As reported in previous literature, PUE@PEG-PE micelles incubation in the presence of 10% FBS at 37 °C for 24 h showed no difference in average size (17.1 nm) or size distribution (Roby et al., 2006). Micelles maintained the same size and showed minimal PUE leakage, which permits to hope that these micelles will be sufficiently stable in the blood within the time intervals allowing for their accumulation in the ischemic zone by the enhanced permeation and retention effect (EPR) (Lukyanov et al., 2004; Dong et al., 2017).

### Core–shell structure of PEG-PE micelles

**Figure 2.**
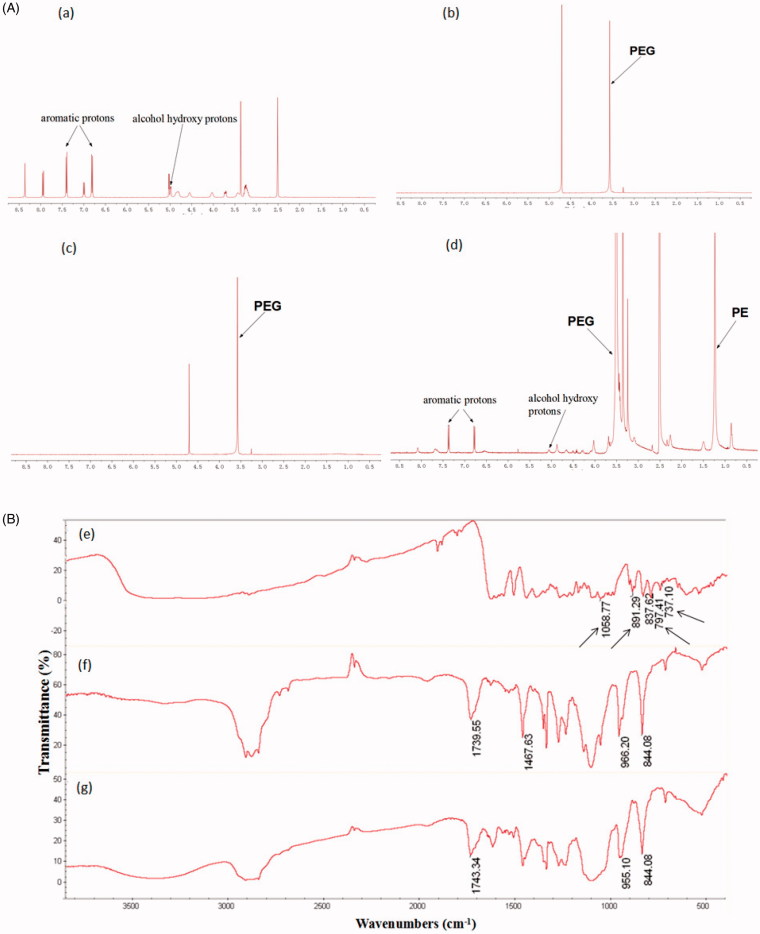
Core–shell structure of PUE@PEG-PE micelles. (A) The ^1^H NMR spectrum of (a) PUE, (b) blank PEG-PE micelle in D_2_O, (c) PUE@PEG-PE micelle in D_2_O, and (d) PUE@PEG-PE micelles in DMSO. (B) FT-IR analysis of (e) PUE, (f) blank PEG-PE micelles, and (g) PUE@PEG-PE micelles.

As shown in Figure 2(A-a), the PUE resonance peaks were observed with characteristic peaks corresponding to alcohol hydroxy protons (4.98, 5.03 ppm) and aromatic protons (6.81, 7.40 ppm). Blank PEG-PE micelles in D_2_O formed a shell structure. As can be seen in the spectrum of Figure 2(A-b), the micelles shells consisting of PEG blocks indicated ^1^H NMR resonance peaks at 3.51 ppm, while the resonances of the PE core were hardly observed because of the insufficient mobility in D_2_O. Similar ^1^H NMR spectrum was observed in the PEG-PE micelles encapsulating PUE in D_2_O. Only PEG resonance peaks were detected in D_2_O whereas PUE peaks were hardly observed. This result clearly confirmed the core–shell structure of micelles (Figure 2(A-c)). When the core-shell structure of PUE@PEG-PE micelles was disrupted in DMSO, PUE was released into the surrounding medium, which was confirmed by the peaks at 5.06 ppm (corresponding to alcohol hydroxy protons of PUE) and at 6.78 and 7.38 ppm (corresponding to the aromatic protons of PUE) in the ^1^H NMR spectra (Figure 2(A-d)). Taken together, these results clearly demonstrated that PUE was intercalated into the PE core of micelles as evident from the absence of peaks corresponding to PUE when analyzed in D_2_O and their appearance when micelles were disrupted in DMSO.

The drug entrapment into the inner core of PEG-PE micelles was also confirmed by the analysis of FT-IR. Figure 2(B) showed the FT-IR spectra of PUE, blank PEG-PE micelles, and PUE@PEG-PE micelles, respectively. The FT-IR spectra of PUE@PEG-PE micelles was basically similar to that of blank PEG-PE micelles. In contrast, the characteristic peaks of PUE at 1058.77, 891.29, 797.41, and 737.10 cm^−1^ were hardly seen in PUE@PEG-PE micelles. This result further indicated that PUE was successfully entrapped into the hydrophobic PE core of polymeric micelles.

### *In vitro* release of PUE from PUE@PEG-PE micelles

**Figure 3.**
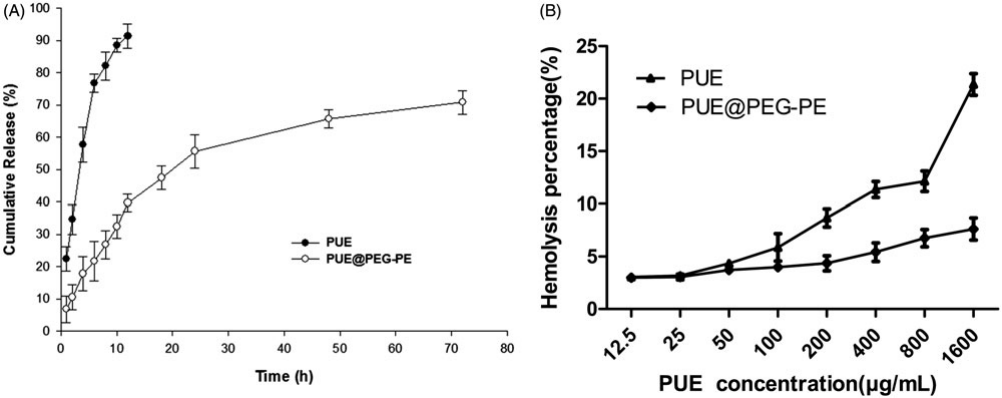
*In vitro* study of PUE@PEG-PE micelles. (A): Release curve of PUE and PUE@PEG-PE micelles. (B): Hemolysis percentages of PUE and PUE@PEG-PE micelles at PUE concentrations ranging from 12.5 to 1600 μg/mL, which were incubated with human red blood cells (RBCs) at 37 °C for 2 h (*n* = 3).

Free PUE was released rapidly, more than 90% of the drug has been released within the first 12 h. In contrast, PUE@PEG-PE micelles exhibited a slight burst release during the first 16 h, followed by a slow and continuous release. Only approximately 70% of PUE was slowly released from the PUE@PEG-PE micelles even after 72 h (Figure 3(A)). These results suggest that PUE@PEG-PE micelles show relatively low leakage at 37 °C and pH 7.4. This kind of release profile is desired to maintain a sustained high drug concentration at the site of action. Since the PUE release is quite slow, it indicates that the core of the PUE@PEG-PE micelles is stiff and glassy (Torchilin, 2005), which confers less mobility to the incorporated drug as compared to mobile cores (Gill et al., 2011). Therefore, the PUE encapsulated in the inner core of PEG-PE micelles was released in a sustained manner for long circulation time.

### Hemolytic assay

The PUE exhibited a dose-dependent hemolytic activity which increased rapidly with the increase of PUE concentration. The hemolysis percentage of PUE reached 21.4% at the PUE concentration of 1600 μg/mL, whereas the hemolysis percentage of PUE@PEG-PE micelles was consistently below 7.6% at PUE concentration varying from 12.5 to 1600 μg/mL (Figure 3(B)). Thus, it can be inferred that PUE@PEG-PE micelles had superiority in hemocompatibility and could be regarded as suitable for intravenous administration. There were two possible explanations for this phenomenon. PUE could be almost entirely surrounded by PEG-PE. Thus, it could luckily inhibit the interaction of RBCs with PUE by the effect of steric shielding or coating of the core–shell structure of PEG-PE micelles, which act as protective barrier against the biological components (Shao et al., 2010). In addition, PEG-PE micelles could also confer its excellent hemocompatibility upon PUE and its special characteristic of long-term sustained release could also decrease the accumulation of the high concentration of PUE (Liu et al., 2016). Therefore, the risk of hemolytic activity of PUE may be reduced dramatically.

### Pharmacokinetics of PUE@PEG-PE micelles in rats

**Figure 4.**
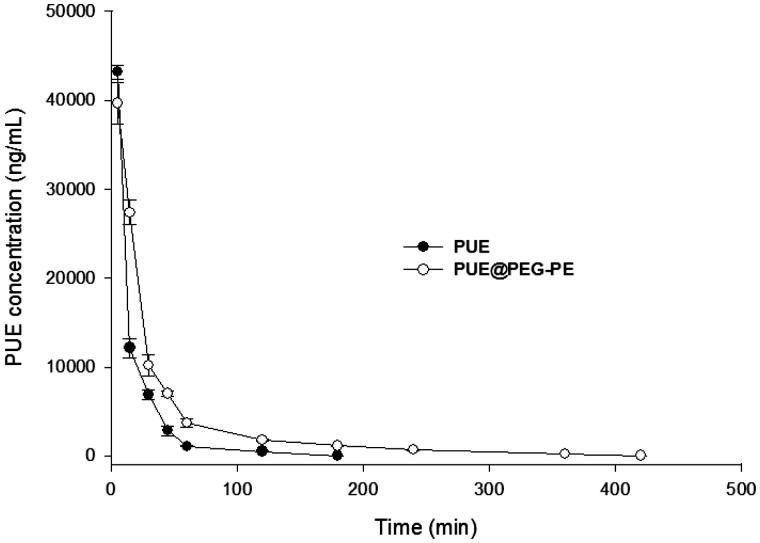
Blood concentration–time profiles in rats after intravenous administration of PUE and PUE@PEG-PE micelles (*n* = 5).

Plasma pharmacokinetics of the free PUE and PUE@PEG-PE micelles were illustrated in Figure 4. The concentration of PUE in circulation decreased with time in healthy rats injected with either PUE or PUE@PEG-PE micelles. PUE was almost undetectable in rat plasma 180 min after free PUE injection, whereas appreciable PUE remained in the plasma of rats treated with PUE@PEG-PE micelles even at 420 min after injection. These results showed that PUE was quickly removed from the circulation system. The average value of AUC_0–∞_was 901,173 ± 10,494 ng/mL/min and the half-life (*t*_1/2_) was about 22.7 ± 1.4 min. Nevertheless, PUE@PEG-PE micelles displayed a relatively slower and steadier drug-release than PUE. PUE loaded in micelles resulted in a *t*_1/2_ of 73.2 ± 2.9 min and AUC_0–∞_ of 1,422,666 ± 44,079 ng/mL/min, which was 3.2 times longer and 1.58 times higher than PUE, respectively. These results further indicated the relatively long persistence of PEG-PE micelles in plasma. This could be explained by the fact that the PEG as the outer shell would reduce the plasma protein adhesion and phagocytosis by RES and produced a long retention in circulation (Gref et al., 1994; Tang et al., 2014). This may help to improve the therapeutic efficacy of encapsulating PUE in micelles (Wang et al., 2016).

### Protective effect of PUE@PEG-PE micelles againstISO-induced H9c2 cells apoptosis

**Figure 5.**
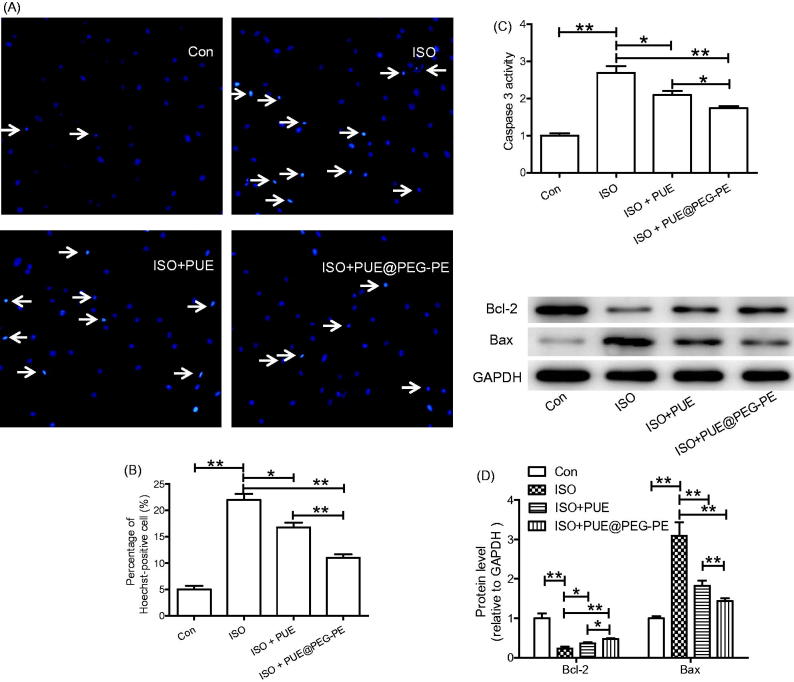
Protective effect of PUE@PEG-PE against ISO-induced H9c2 cells apoptosis. (A, B) Hoechst 33258 staining; (C) Caspase 3 activity; (D) Apoptosis-related protein (Bcl-2 and Bax) expression. Date are mean ± SEM (*n* = 3). (*p < .05, **p < .01).

To assess the protective effect of PUE@PEG-PE micelles against ISO-induced H9c2 cells apoptosis, Hoechst 33258 staining was performed. As shown in Figure 5(A,B), ISO increased the number of Hoechst positive cells with condensed and fragmented fluorescent nuclei, which was inhibited by pretreatment with PUE or PUE@PEG-PE micelles. Moreover, compared with PUE, pretreatment with PUE@PEG-PE micelles can further decrease the Hoechst positive cells.

Since increased caspase 3 activity and abnormal expression of apoptosis-related proteins have been shown to play key roles in cells apoptosis, caspase 3 activity and apoptosis-related protein expression (Bcl-2 and Bax) were determined (Li et al., 2009; Ruan et al., 2012; Peng et al., 2015; Ouyang et al., 2016). Results showed that ISO induced the increase of caspase 3 activity, Bax expression, and decrease of Bcl-2 expression. Both PUE and PUE@PEG-PE micelles inhibited the effect of ISO, and PUE@PEG-PE micelles exhibited stronger effect than PUE (Figure 5(C,D)). These results collectively suggested that PEG-PE micelles can enhance the protective effects of PUE against ISO-induced H9c2 cells apoptosis.

### Cellular uptake of micelles

**Figure 6.**
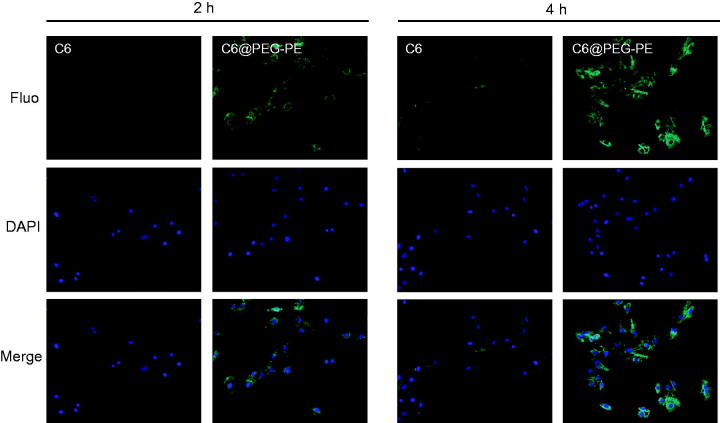
Cellular uptake of C6 and C6@PEG-PE micelles incubation in H9c2 cells at different time. Green shows C6 or C6@PEG-PE micelles, Blue indicates the cell nucleus.

Fluorescence microscope images indicating cellular uptake of micelles in H9c2 cells was presented in Figure 6. H9c2 cells treated with C6 loaded PEG-PE (C6@PEG-PE) micelles, fluorescent intensity appeared to increase with incubation time ranging from 2 to 4 h, whereas no detectable amount of free C6 as indicated by the fluorescent intensity was present in the cell, this result indicated that free C6 was taken up less by H9c2 cells. Similar results have been observed in other literature reports, highly hydrophobic chemicals (e.g. C6) are difficult to enter into cells in an aqueous medium unless a high content of DMSO included in the medium (Rivolta et al., 2011; Hu et al., 2017). After entrapment in PEG-PE micelles, C6 was more likely to be taken up by cells, and intracellular fluorescence intensity was significantly higher than that in the C6 solution. In addition, after 4 h incubation, Figure 6 also showed a high degree of co-localization of C6 in the cells with micelles distributing around the nucleus. These results suggested that PEG-PE micelles can be internalized by H9c2 cells and could also deliver more drugs into the living cells (Xu et al., 2017). The possible reason is that owing to the surface-active property of amphiphilic nature, PEG-PE micelles insert into the cell membrane, induce an increase in the mobility of cell membrane and accelerate the flip of C6 across the membrane (Demina et al., 2005). Higher membrane fluidity of H9c2 cells after incubation with PEG-PE micelles is powerfully helpful for encapsulating drug trafficking across the cell membrane.

To confirm whether the enhanced anti-apoptotic effect of PUE@PEG-PE micelles was due to the more drugs trafficking across the cell membrane. Taking into account that intracellular drug concentration detection is relatively difficult, and the need to use the sophisticated and expensive LC/MS-MS. The PUE concentration in the extracellular medium was measured. The initial drug concentration minus the extracellular drug concentration can reflect the intracellular drug concentration. As shown in Supplementary Figure S1. Overall, the concentration of extracellular drugs in both groups showed a decreasing trend over time, indicating that the reduced drug entered into the H9c2 cells. The concentration of extracellular drugs in the PUE@PEG-PE micelles was significantly lower than that in the PUE. These results indicated that more drugs were carried by the PEG-PE micelles and transported into the H9c2 cells. With the increase of intracellular drug concentration, the efficacy will be increased accordingly, and this may explain why the PEG-PE micelles encapsulating PUE have an enhanced anti-apoptotic effect compared with free PUE *in vitro*.

## Conclusions

In the present study, PEG-PE micelles were able to effectively encapsulate PUE with entrapment efficiency of 68.7% in nanoparticle size range (16 nm). The *in vitro* release studies indicated a sustained release potential of the PEG-PE micelles. Pharmacokinetic studies in SD rats suggested the extended circulation time and improved bioavailability of the PUE@PEG-PE micelles. Compared with the free PUE, PUE@PEG-PE micelles exhibited better protective effect against ISO-induced H9c2 cells apoptosis, which may be attributed to the high cellular uptake ability of PEG-PE micelles as confirmed by the comparative study of free C6 and C6@PEG-PE micelles. The results of the extracellular drug concentration test also indirectly confirmed that intracellular drug concentration of the PUE@PEG-PE micelles was significantly higher than that of the PUE. These results collectively indicated that the PEG-PE micelles were promising nanocarriers for efficient delivery of cardiovascular drugs and other therapeutic agents. In view of the night attack of cardiovascular disease, abnormal apoptotic necrosis of cardiomyocytes often happens, it is particularly important to maintain effective blood concentrations for a longer period of time. PUE@PEG-PE micelles with the advantage of slow release and excellent anti-apoptotic effect may lay the experimental foundation for solving these problems. In addition, this sustained-release delivery system can also reduce the number of administrations, especially for chronic cardiovascular diseases requiring long-term medication. Further study on drug efficacy and safety of PUE@PEG-PE micelles was underway to evaluate whether the superior anti-apoptotic effect and better pharmacokinetic profile of PUE were sufficient for the treatment of cardiovascular diseases. Specifically, an estimate of the required dose of the drug and how much micelles would be needed for a specific cardiovascular disease should be based on a large number of preclinical and clinical data.

## Supplementary Material

IDRD_liu_et_al_Supplemental_Content.doc

## References

[CIT0001] Abouzeid AH, Patel NR, Torchilin VP. (2014). Polyethylene glycol-phosphatidylethanolamine (PEG-PE)/vitamin E micelles for co-delivery of paclitaxel and curcumin to overcome multi-drug resistance in ovarian cancer. Int J Pharm 464:178–84. 24440402 10.1016/j.ijpharm.2014.01.009 PMC3980878

[CIT0002] Chen M, Zhou X, Yu L, et al (2016). Low-level vagus nerve stimulation attenuates myocardial ischemic reperfusion injury by antioxidative stress and antiapoptosis reactions in canines. J Cardiovasc Electrophysiol 27:224–31. 26546374 10.1111/jce.12850

[CIT0003] Cui Y, Dong H, Cai X, et al (2012). Mesoporous silica nanoparticles capped with disulfide-linked PEG gatekeepers for glutathione-mediated controlled release. ACS Appl Mater Interfaces 4:3177–83. 22646097 10.1021/am3005225

[CIT0004] Dabholkar RD, Sawant RM, Mongayt DA, et al (2006). Polyethylene glycol-phosphatidylethanolamine conjugate (PEG-PE)-based mixed micelles: some properties, loading with paclitaxel, and modulation of P-glycoprotein-mediated efflux. Int J Pharm 315:148–57. 16616818 10.1016/j.ijpharm.2006.02.018

[CIT0005] Demina T, Grozdova I, Krylova O, et al (2005). Relationship between the structure of amphiphilic copolymers and their ability to disturb lipid bilayers. Biochemistry 44:4042–54. 15751981 10.1021/bi048373q

[CIT0006] Dong Z, Guo J, Xing X, et al (2017). RGD modified and PEGylated lipid nanoparticles loaded with puerarin: formulation, characterization and protective effects on acute myocardial ischemia model. Biomed Pharmacother 89:297–304. 28236703 10.1016/j.biopha.2017.02.029

[CIT0007] Endres TK, Beck-Broichsitter M, Samsonova O, et al (2011). Self-assembled biodegradable amphiphilic PEG-PCL-lPEI triblock copolymers at the borderline between micelles and nanoparticles designed for drug and gene delivery. Biomaterials 32:7721–31. 21782238 10.1016/j.biomaterials.2011.06.064

[CIT0008] Gill KK, Nazzal S, Kaddoumi A. (2011). Paclitaxel loaded PEG(5000)-DSPE micelles as pulmonary delivery platform: formulation characterization, tissue distribution, plasma pharmacokinetics, and toxicological evaluation. Eur J Pharm Biopharm 79:276–84. 21575719 10.1016/j.ejpb.2011.04.017

[CIT0009] Gref R, Minamitake Y, Peracchia MT, et al (1994). Biodegradable long-circulating polymeric nanospheres. Science 263:1600–3. 8128245 10.1126/science.8128245

[CIT0010] Hou SZ, Su ZR, Chen SX, et al (2011). Role of the interaction between puerarin and the erythrocyte membrane in puerarin-induced hemolysis. Chem Biol Interact 192:184–92. 21453687 10.1016/j.cbi.2011.03.007

[CIT0011] Hu X, Yang FF, Liu CY, et al (2017). In vitro uptake and transport studies of PEG-PLGA polymeric micelles in respiratory epithelial cells. Eur J Pharm Biopharm 114:29–37. 28093351 10.1016/j.ejpb.2017.01.004

[CIT0012] Hu X, Yang FF, Quan LH, et al (2014). Pulmonary delivered polymeric micelles – pharmacokinetic evaluation and biodistribution studies. Eur J Pharm Biopharm 88:1064–75. 25460153 10.1016/j.ejpb.2014.10.010

[CIT0013] Huang F, Liu K, Du H, et al (2012). Puerarin attenuates endothelial insulin resistance through inhibition of inflammatory response in an IKKbeta/IRS-1-dependent manner. Biochimie 94:1143–50. 22314193 10.1016/j.biochi.2012.01.018

[CIT0014] Kohay H, Sarisozen C, Sawant R, et al (2017). PEG-PE/clay composite carriers for doxorubicin: effect of composite structure on release, cell interaction and cytotoxicity. Acta Biomater 55:443–54. 28400314 10.1016/j.actbio.2017.04.008

[CIT0015] Lazarjani HA, Vasheghani-Farahani E, Barani L, et al (2010). Effect of polymer concentration on camouflaging of pancreatic islets with mPEG-succinimidyl carbonate. Artif Cells Blood Substit Immobil Biotechnol 38:250–8. 20486872 10.3109/10731199.2010.488634

[CIT0016] Li JQ, Qi HZ, He ZJ, et al (2009). Cytoprotective effects of human interleukin-10 gene transfer against necrosis and apoptosis induced by hepatic cold ischemia/reperfusion injury. J Surg Res 157:e71–8. 19555976 10.1016/j.jss.2009.03.004

[CIT0017] Li WH, Lu M, Zhang YH, et al (2017). Puerarin attenuates the daunorubicin-induced apoptosis of H9c2 cells by activating the PI3K/Akt signaling pathway via the inhibition of Ca^2+^ influx. Int J Mol Med 40:1889–94. 29039532 10.3892/ijmm.2017.3186

[CIT0018] Liu B, Wu ZY, Li YP, et al (2015). Puerarin prevents cardiac hypertrophy induced by pressure overload through activation of autophagy. Biochem Biophys Res Commun 464:908–15. 26188094 10.1016/j.bbrc.2015.07.065

[CIT0019] Liu CM, Ma JQ, Sun YZ. (2011). Protective role of puerarin on lead-induced alterations of the hepatic glutathione antioxidant system and hyperlipidemia in rats. Food Chem Toxicol 49:3119–27. 22001170 10.1016/j.fct.2011.09.007

[CIT0020] Liu CM, Ma JQ, Sun YZ. (2012). Puerarin protects rat kidney from lead-induced apoptosis by modulating the PI3K/Akt/eNOS pathway. Toxicol Appl Pharmacol 258:330–42. 22172631 10.1016/j.taap.2011.11.015

[CIT0021] Liu LJ, Liu LQ, Bo T, et al (2013). Puerarin suppress apoptosis of human osteoblasts via ERK signaling pathway. Int J Endocrinol 2013:786574. 23843790 10.1155/2013/786574 PMC3694486

[CIT0022] Liu X, Ding Y, Zhao B, et al (2016). In vitro and in vivo evaluation of puerarin-loaded PEGylated mesoporous silica nanoparticles. Drug Dev Ind Pharm 42:2031–7. 27282345 10.1080/03639045.2016.1190742

[CIT0023] Lu Q, Xiang DX, Yuan HY, et al (2014). Puerarin attenuates calcification of vascular smooth muscle cells. Am J Chin Med 42:337–47. 24707866 10.1142/S0192415X14500220

[CIT0024] Lu X, Zhang F, Qin L, et al (2010). Polymeric micelles as a drug delivery system enhance cytotoxicity of vinorelbine through more intercellular accumulation. Drug Deliv 17:255–62. 20307251 10.3109/10717541003702769

[CIT0025] Lukyanov AN, Hartner WC, Torchilin VP. (2004). Increased accumulation of PEG-PE micelles in the area of experimental myocardial infarction in rabbits. J Control Release 94:187–93. 14684282 10.1016/j.jconrel.2003.10.008

[CIT0026] Luo CF, Yuan M, Chen MS, et al (2011). Determination of puerarin in rat plasma by rapid resolution liquid chromatography tandem mass spectrometry in positive ionization mode. J Chromatogr B Analyt Technol Biomed Life Sci 879:1497–501. 10.1016/j.jchromb.2011.03.035 21511546

[CIT0027] Musacchio T, Vaze O, D'souza G, Torchilin VP. (2010). Effective stabilization and delivery of siRNA: reversible siRNA-phospholipid conjugate in nanosized mixed polymeric micelles. Bioconjugate Chem 21:1530–6. 10.1021/bc100199c 20669936

[CIT0028] Ouyang F, Huang H, Zhang M, et al (2016). HMGB1 induces apoptosis and EMT in association with increased autophagy following H/R injury in cardiomyocytes. Int J Mol Med 37:679–89. 26847839 10.3892/ijmm.2016.2474 PMC4771104

[CIT0029] Peng J, Li X, Zhang D, et al (2015). Hyperglycemia, p53, and mitochondrial pathway of apoptosis are involved in the susceptibility of diabetic models to ischemic acute kidney injury. Kidney Int 87:137–50. 24963915 10.1038/ki.2014.226 PMC4276728

[CIT0030] Raza K, Kumar N, Misra C, et al (2016). Dextran-PLGA-loaded docetaxel micelles with enhanced cytotoxicity and better pharmacokinetic profile. Int J Biol Macromol 88:206–12. 27037052 10.1016/j.ijbiomac.2016.03.064

[CIT0031] Rivolta I, Panariti A, Lettiero B, et al (2011). Cellular uptake of coumarin-6 as a model drug loaded in solid lipid nanoparticles. J Physiol Pharmacol 62:45–53. 21451209

[CIT0032] Roby A, Erdogan S, Torchilin VP. (2006). Solubilization of poorly soluble PDT agent, meso-tetraphenylporphin, in plain or immunotargeted PEG-PE micelles results in dramatically improved cancer cell killing in vitro. Eur J Pharm Biopharm 62:235–40. 16326084 10.1016/j.ejpb.2005.09.010 PMC1634738

[CIT0100] Ruan W, Xu JM, Li SB, et al (2012). Effects of down-regulation of microRNA-23a on TNF-α-induced endothelial cell apoptosis through caspase-dependent pathways. Cardiovasc Res 93:623–32. 22038739 10.1093/cvr/cvr290

[CIT0033] Sarisozen C, Vural I, Levchenko T, et al (2012a). Long-circulating PEG-PE micelles co-loaded with paclitaxel and elacridar (GG918) overcome multidrug resistance. Drug Deliv 19:363–70. 23030458 10.3109/10717544.2012.724473

[CIT0034] Sarisozen C, Vural I, Levchenko T, et al (2012b). PEG-PE-based micelles co-loaded with paclitaxel and cyclosporine A or loaded with paclitaxel and targeted by anticancer antibody overcome drug resistance in cancer cells. Drug Deliv 19:169–76. 22506922 10.3109/10717544.2012.674163

[CIT0035] Sawant RR, Sawant RM, Torchilin VP. (2008). Mixed PEG-PE/vitamin E tumor-targeted immunomicelles as carriers for poorly soluble anti-cancer drugs: improved drug solubilization and enhanced in vitro cytotoxicity. Eur J Pharm Biopharm 70:51–7. 18583114 10.1016/j.ejpb.2008.04.016 PMC2603419

[CIT0036] Sawant RR, Torchilin VP. (2010). Multifunctionality of lipid-core micelles for drug delivery and tumour targeting. Mol Membr Biol 27:232–46. 20929339 10.3109/09687688.2010.516276

[CIT0037] Shao K, Huang R, Li J, et al (2010). Angiopep-2 modified PE-PEG based polymeric micelles for amphotericin B delivery targeted to the brain. J Control Release 147:118–26. 20609375 10.1016/j.jconrel.2010.06.018

[CIT0038] Szilagyi I, Trefalt G, Tiraferri A, et al (2014). Polyelectrolyte adsorption, interparticle forces, and colloidal aggregation. Soft Matter 10:2479–502. 24647366 10.1039/c3sm52132j

[CIT0039] Tang J, Wang X, Wang T, et al (2014). In vivo pharmacokinetics, biodistribution and antitumor effect of amphiphilic poly(L-amino acids) micelles loaded with a novel all-trans retinoic acid derivative. Eur J Pharm Sci 51:157–64. 24076464 10.1016/j.ejps.2013.09.016

[CIT0040] Tong S, Hou S, Ren B, et al (2011). Self-assembly of phospholipid-PEG coating on nanoparticles through dual solvent exchange. Nano Lett 11:3720–6. 21793503 10.1021/nl201978c PMC3173588

[CIT0041] Torchilin VP. (2005). Lipid-core micelles for targeted drug delivery. Curr Drug Deliv 2:319–27. 16305435 10.2174/156720105774370221

[CIT0042] Wang J, Wang Y, Liang W. (2012). Delivery of drugs to cell membranes by encapsulation in PEG-PE micelles. J Control Release 160:637–51. 22405904 10.1016/j.jconrel.2012.02.021

[CIT0043] Wang Q, Jiang J, Chen W, et al (2016). Targeted delivery of low-dose dexamethasone using PCL-PEG micelles for effective treatment of rheumatoid arthritis. J Control Release 230:64–72. 27057749 10.1016/j.jconrel.2016.03.035

[CIT0044] Wang Y, Fan W, Dai X, et al (2014). Enhanced tumor delivery of gemcitabine via PEG-PE/TPGS mixed micelles. Mol Pharm 11:1140–50. 24579673 10.1021/mp4005904 PMC3993932

[CIT0045] Wong KH, Li GQ, Li KM, et al (2011). Kudzu root: traditional uses and potential medicinal benefits in diabetes and cardiovascular diseases. J Ethnopharmacol 134:584–607. 21315814 10.1016/j.jep.2011.02.001

[CIT0046] Wu J, Zhang X, Zhang B. (2014). Efficacy and safety of puerarin injection in treatment of diabetic peripheral neuropathy: a systematic review and meta-analysis of randomized controlled trials. J Tradit Chin Med 34:401–10. 25185357 10.1016/s0254-6272(15)30039-x

[CIT0047] Xu Y, Wang S, Chan HF, et al (2017). Triphenylphosphonium-modified poly(ethylene glycol)-poly(epsilon-caprolactone) micelles for mitochondria- targeted gambogic acid delivery. Int J Pharm 522:21–33. 28215509 10.1016/j.ijpharm.2017.01.064

[CIT0048] Yassin MA, Appelhans D, Wiedemuth R, et al (2015). Overcoming concealment effects of targeting moieties in the PEG corona: controlled permeable polymersomes decorated with folate-antennae for selective targeting of tumor cells. Small 11:1580–91. 25363281 10.1002/smll.201402581

[CIT0049] Yuan Y, Zong J, Zhou H, et al (2014). Puerarin attenuates pressure overload-induced cardiac hypertrophy. J Cardiol 63:73–81. 23906530 10.1016/j.jjcc.2013.06.008

[CIT0050] Zhang H, Zhang L, Zhang Q, et al (2011). Puerarin: a novel antagonist to inward rectifier potassium channel (IK1). Mol Cell Biochem 352:117–23. 21327545 10.1007/s11010-011-0746-0

