# Supplementary material for: Puerarin-loaded PEG-PE micelles with enhanced anti-apoptotic effect and better pharmacokinetic profile
Source: Drug Deliv. 2018 Mar 28;25(1):827–37. doi: 10.1080/10717544.2018.1455763 (PMC6058490; doi:10.1080/10717544.2018.1455763)
Supplement: IDRD_liu_et_al_Supplemental_Content.doc [file IDRD_A_1455763_SM9949.doc]

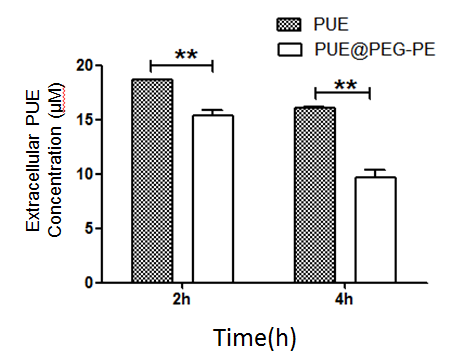


**Figure S1.** Extracellular PUE concentration of H9c2 cells incubating with PUE and PUE @PEG-PE micelles at different time (n = 3). (********p* < 0.05, *********p* < 0.01).
